# Beclomethasone Has Lesser Suppressive Effects on Inflammation and Antibacterial Immunity Than Fluticasone or Budesonide in Experimental Infection Models

**DOI:** 10.1016/j.chest.2020.05.531

**Published:** 2020-05-23

**Authors:** Faisal Kamal, Nicholas Glanville, Wangmingyu Xia, Eteri Bakhsoliani, Julia Aniscenko, Nathan W. Bartlett, Michael R. Edwards, Sebastian L. Johnston, Aran Singanayagam

**Affiliations:** a National Heart and Lung Institute, Imperial College, London, UK; b Faculty of Health and Medicine and Priority Research Centre for Healthy Lungs, University of Newcastle, Australia

To the Editor:

Inhaled corticosteroids (ICS) are mainstay therapies in COPD but are consistently linked with increased pneumonia susceptibility. There is speculation regarding possible intraclass differences in pneumonia risk between ICS agents, with some studies suggesting that budesonide and beclomethasone dipropionate (BDP) confer lower pneumonia risk than fluticasone propionate (FP).^1^, ^2^, ^3^ This has not been consistently shown^4^^,^^5^ and remains controversial. In the absence of head-to-head comparator trials, it is impossible to conclusively ascertain intraclass differences in pneumonia propensity. No previous studies have compared the relative potential of these three ICS agents to impair host defense in experimental infection models, and mechanisms underlying any potential differential effects on pneumonia susceptibility are unknown. The agents differ in terms of glucocorticoid receptor affinity, solubility, and antiinflammatory potency,^6^ and thus they may have differing abilities to impair critical components of antimicrobial host defense. We have recently reported that FP can impair epithelial control of the pneumonia-causing pathogen *Streptococcus pneumoniae,* mechanistically through inhibition of the antimicrobial peptide (AMP) cathelicidin.^7^ Using experiments in human cells and mouse infection models, we performed a head-to-head comparison of the effects of the major ICS agents used in COPD on innate immunity.

## Methods

BEAS2B airway epithelial cells (AECs) were treated with FP, budesonide, and BDP (Sigma-Aldrich) at 0.1- to 1,000-nM concentrations or vehicle dimethyl sulfoxide in 10% RPMI medium at 37°C for 1 hour before *S pneumoniae* D39 infection (1 × 10^6^ CFU/mL).^7^ In separate studies, 6-week-old female wild-type C57BL/6 mice were treated intranasally under isofluorane anesthesia with 20 μg FP, budesonide, BDP, or vehicle and infected with 5 × 10^5^ CFU *S pneumoniae* D39 or PBS control.^7^ Pneumococcal loads were quantified in homogenized lung tissue or cell supernatants and immune or inflammatory mediators measured in BAL or cell supernatants by enzyme-linked immunosorbent assay (ELISA). For in vitro experiments, conditions were run in triplicate wells and experiments repeated three times independently, with mean data from combined experiments analyzed by one-way analysis of variance with Bonferroni’s multiple comparison test with use of the Brown Forsythe test to confirm equality of group variances. Experiments in mice involved five animals/group, and data shown are representative of two independent experiments, analyzed using Kruskal-Wallis test with Dunn’s multiple comparison test.

## Results

**Figure 1:**
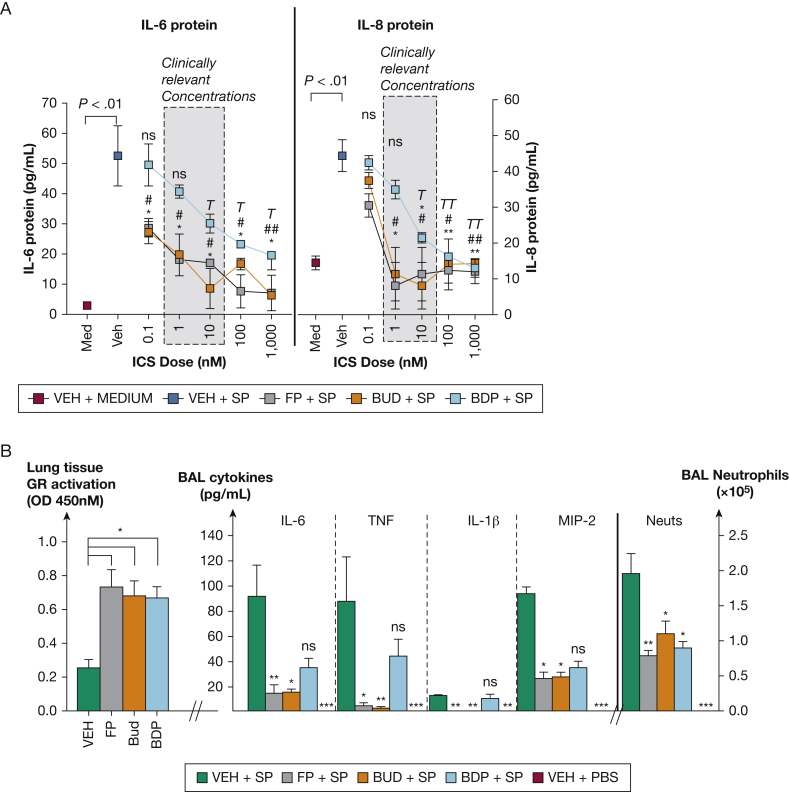
Comparison of antiinflammatory effects of ICS agents during bacterial infection in vitro and in vivo. A, BEAS2B bronchial epithelial cells were treated with fluticasone propionate (FP), budesonide (BUD), beclomethasone dipropionate (BDP), or vehicle (VEH) at 0.1-1,000 nM concentrations and infected with Streptococcus pneumoniae D39. IL-6 and CXCL8/IL-8 protein was measured in cell supernatants by enzyme-linked immunosorbent assay (ELISA). B, C57BL/6 mice were treated intranasally with FP, budesonide, or beclomethasone and infected with S pneumoniae D39 or phosphate-buffered saline (PBS) control. Glucocorticoid receptor activation in lung tissue was assessed by measuring nuclear DNA binding by ELISA. Pro-inflammatory cytokines IL-6, tumor necrosis factor, and IL-1β and neutrophil chemokine CXCL2/MIP-2 were measured in BAL at 8 hours post-infection by ELISA. BAL neutrophils at 24 hours post-infection were enumerated using cytospins. Data shown as mean (±SEM) for n = 3 independent experiments combined in A or n = 5-8 mice/group representative of 2 independent experiments in B. Data analyzed by one-way analysis of variance with Bonferroni post-test. Statistical significance shown in comparison with vehicle + S pneumoniae (VEH+SP). A, ∗corrected P < .05 and ∗∗corrected P < .01 (FP + SP); #corrected P < .05 and ##corrected P < .01 (BUD + SP); TP < .05, TT P < .01 (BDP + SP) group. B, ∗corrected P < .05; ∗∗corrected P < .01; ∗∗∗corrected P < .001. NS = nonsignificant.

We initially evaluated antiinflammatory effects of equimolar concentrations (0.1-1,000 nM) of FP, budesonide, and BDP in AECs infected with *S pneumoniae*. Previous studies of ICS administration in humans show that a single inhaled dose of FP results in approximately 10 nM lung tissue concentration.^8^ Because FP is reported to be approximately twofold more potent than budesonide or BDP,^6^ 1- and 10-nM doses capture the full range of clinically relevant tissue concentrations of all three agents encountered in vivo. FP and budesonide (at concentrations of 1 nM and higher) suppressed induction of the pro-inflammatory cytokines IL-6 and chemokine (C-X-C motif) ligand 8/IL-8, with BDP only having significant effects at 10 nM or higher (Fig 1A). To confirm these effects in vivo, 20 μg of each ICS was administered in *S pneumoniae*-infected mice. There was no difference in lung glucocorticoid receptor activation between the three agents at this dose (Fig 1B). Significant suppression of IL-6, tumor necrosis factor, and IL-1β and the neutrophil chemokine CXCL2/MIP-2 was only observed for FP and budesonide (Fig 1B**).** All three ICS significantly reduced airway neutrophil recruitment (Fig 1B). Combined, these data indicate that FP and budesonide and, to a lesser extent, BDP can suppress inflammation during bacterial infection.

**Figure 2:**
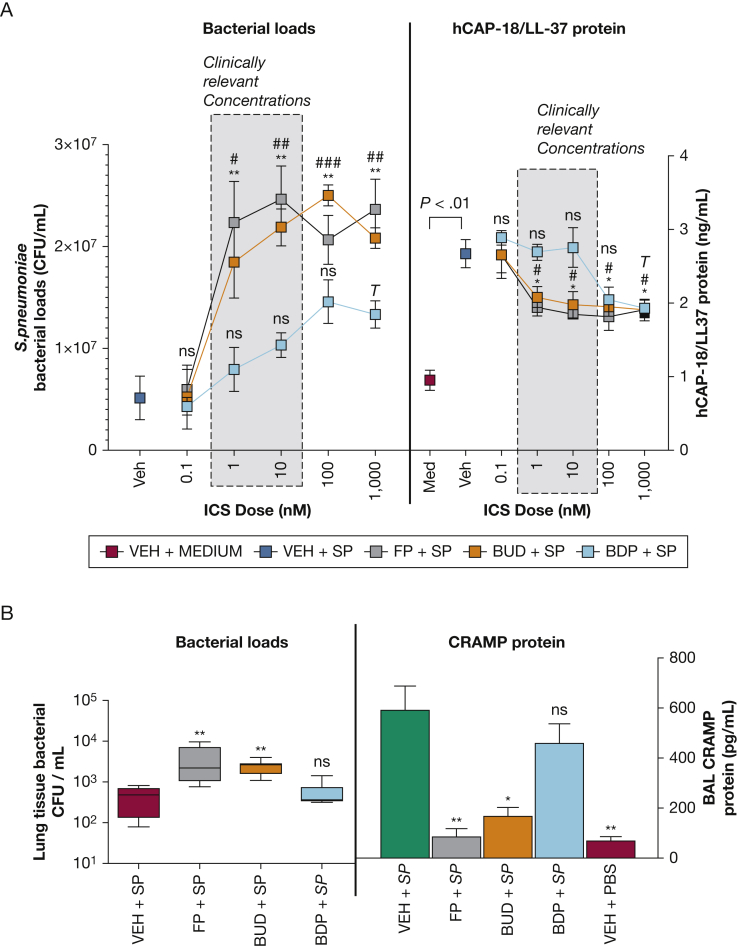
Beclomethasone dipropionate has lesser effects on antibacterial immunity and bacterial replication than fluticasone propionate or budesonide. A, BEAS2B bronchial epithelial cells were treated with fluticasone propionate (FP), budesonide (BUD), beclomethasone dipropionate (BDP) at 0.1-1,000 nM concentrations or vehicle (VEH) control and infected with Streptococcus pneumoniae D39. Pneumococcal bacterial loads were measured by quantitative culture at 24 hours. hCAP18/LL-37 protein was measured in cell supernatants at 8 hours by enzyme-linked immunosorbent assay (ELISA). B, C57BL/6 mice were intranasally treated with FP, budesonide, or beclomethasone and infected with S pneumoniae D39 or phosphate-buffered saline (PBS) control. Lung bacterial loads were measured by quantitative culture at 8 hours. Cathelicidin-related antimicrobial peptide (CRAMP) was measured in BAL at 8 hours by ELISA. Data shown as mean (±SEM) for n = 3 independent experiments combined in A or n = 5-8 mice/group representative of 2 independent experiments in B. Data analyzed by one-way analysis of variance with Bonferroni post-test. Statistical significance shown in comparison with vehicle + S pneumoniae (VEH+SP) group. A, ∗corrected P < .05 and ∗∗corrected P < .01 (FP + SP); #corrected P < .05 and ##corrected P < .01, ###corrected P < .001 (BUD + SP); T corrected P < .05 (BDP + SP) group. B, ∗corrected P < .05 and ∗∗ corrected P < .01. ELISA = enzyme-linked immunosorbent assay. See Figure 1 legend for expansion of other abbreviation.

We next evaluated effects of ICS on bacterial burden in AECs. FP and budesonide, at concentrations of 1 nM or higher, increased bacterial loads at 24 hours, with significant effects only observed for BDP at the highest concentration (1,000 nM) (Fig 2A). Previously, we reported that FP increases bacterial loads via suppression of the antimicrobial peptide (AMP) cathelicidin, an effect that occurs independently of other immune suppression and likely mediates ICS-related pneumonia risk in COPD.^7^ Accordingly, FP and budesonide suppressed epithelial human cathelicidin antimicrobial protein-18/LL-37 (hCAP-18/LL-37) induction by *S pneumoniae* at concentrations of 1 nM or higher, whereas BDP again only had effects at the maximal 1,000-nM concentration (Fig 2A). Similar effects were observed in vivo, with increased lung bacterial loads and reduced induction of the cathelicidin-related AMP (mouse ortholog) observed when FP or budesonide but not BDP was administered in *S pneumoniae*-infected mice (Fig 2B).

## Discussion

Our experimental studies indicate an intra-class differential effect of ICS agents on antibacterial immunity. All three ICS agents could suppress inflammation during bacterial infection; however, at clinically relevant concentrations of 1 and 10 nM, only FP and budesonide inhibited cathelicidin (an AMP implicated in ICS-related pneumonia^7^) and increased bacterial loads. Because BDP also exhibited lesser antiinflammatory effects, this effect may be related to an overall lower potency to impair innate mediator production during pathogenic infection. Our data are supported by findings from a recent trial showing that addition of beclomethasone did not increase pneumonia frequency compared with dual bronchodilator therapy alone.^3^

Some clinical studies have reported that budesonide has lesser effects on pneumonia risk than FP,^1^^,^^2^ with in vitro studies suggesting that this may be related to differential effects on bacterial adhesion^9^ or receptor expression.^10^ We observed no difference in the ability of FP and budesonide to impair cathelicidin and increase bacterial burden. In contrast to these other studies, which have solely used in vitro experiments, we report equivalent effects of FP and budesonide both in vitro and in vivo. Unlike some studies,^9^ we did not attempt to adjust for dose equivalence between the three ICS in our experimental models, because it is extremely difficult to accurately recapitulate differences in effective doses/complexities of inhaled human drug administration between individual ICS agents in which pharmacokinetics may be affected by several factors, including inhaler device, particle size, aqueous solubility, and epithelial permeability.^6^ We instead examined a full concentration range for all three agents to capture the full spectrum of doses and, in vitro, show that a 100-fold higher dose of BDP (which far exceeds the reported approximately 2:1 equivalence ratio) still failed to have comparable effects to a 1-nM dose of FP or budesonide, thus clearly showing a lesser potential for BDP to impair antibacterial immunity. Our data therefore indicate that BDP potentially has lesser beneficial antiinflammatory effects but conversely has reduced potential to impart the detrimental effect of inhibiting protective antimicrobial responses.

We have previously reported that effects of FP on cathelicidin and bacterial replication occur consistently in healthy or diseased (COPD) models.^7^ Our studies here were conducted in nondiseased airway epithelial cells and mouse infection models. Whether similar differences between ICS agents occur in smoke exposure animal models or primary cells from patients with COPD, the group at greatest risk of developing ICS-related pneumonia, remains to be seen*.* COPD patients are most commonly treated with ICS combined with long-acting bronchodilators; future studies should evaluate effects of clinically relevant combination therapies and also assess other bacterial pathogens of importance in COPD such as *Haemophilus influenzae.* Head-to-head clinical trials of different ICS agents examining effects on immune mediators, microbiota, and pneumonia development will ultimately be required to confirm that these findings are relevant to human disease.
